# Identification of Candidate Genes and Pathways Associated with Obesity-Related Traits in Canines via Gene-Set Enrichment and Pathway-Based GWAS Analysis

**DOI:** 10.3390/ani10112071

**Published:** 2020-11-09

**Authors:** Sunirmal Sheet, Srikanth Krishnamoorthy, Jihye Cha, Soyoung Choi, Bong-Hwan Choi

**Affiliations:** Animal Genome & Bioinformatics, National Institute of Animal Science, RDA, Wanju 55365, Korea; sunirmal.micro@gmail.com (S.S.); kris87@korea.kr (S.K.); wischa91@korea.kr (J.C.); csy7pp@korea.kr (S.C.)

**Keywords:** obesity, post-GWAS, gene-set enrichment, pathway analysis, functional annotation, genetic variants, blood sugar, body weight, canine

## Abstract

**Simple Summary:**

Obesity is a serious health issue and is increasing at an alarming rate in several dog breeds, but there is limited information on the genetic mechanism underlying it. Moreover, there have been very few reports on genetic markers associated with canine obesity. These studies were limited to the use of a single breed in the association study. In this study, we have performed a GWAS and supplemented it with gene-set enrichment and pathway-based analyses to identify causative loci and genes associated with canine obesity in 18 different dog breeds. From the GWAS, the significant markers associated with obesity-related traits including body weight (*CACNA1B*, *C22orf39*, *U6*, *MYH14*, *PTPN2*, *SEH1L*) and blood sugar (*PRSS55*, *GRIK2*), were identified. Furthermore, the gene-set enrichment and pathway-based analysis (GESA) highlighted five enriched pathways (Wnt signaling pathway, adherens junction, pathways in cancer, axon guidance, and insulin secretion) and seven GO terms (fat cell differentiation, calcium ion binding, cytoplasm, nucleus, phospholipid transport, central nervous system development, and cell surface) which were found to be shared among all the traits.

**Abstract:**

The present study aimed to identify causative loci and genes enriched in pathways associated with canine obesity using a genome-wide association study (GWAS). The GWAS was first performed to identify candidate single-nucleotide polymorphisms (SNPs) associated with obesity and obesity-related traits including body weight and blood sugar in 18 different breeds of 153 dogs. A total of 10 and 2 SNPs were found to be significantly (*p* < 3.74 × 10^−7^) associated with body weight and blood sugar, respectively. None of the SNPs were identified to be significantly associated with obesity trait. We subsequently followed up the GWAS analysis with gene-set enrichment and pathway analyses. A gene-set with 1057, 1409, and 1243 SNPs annotated to 449, 933 and 820 genes for obesity, body weight, and blood sugar, respectively was created by sub-setting the GWAS result at a threshold of *p* < 0.01 for the gene-set enrichment analysis. In total, 84 GO and 21 KEGG pathways for obesity, 114 GO and 44 KEGG pathways for blood sugar, 120 GO and 24 KEGG pathways for body weight were found to be enriched. Among the pathways and GO terms, we highlighted five enriched pathways (Wnt signaling pathway, adherens junction, pathways in cancer, axon guidance, and insulin secretion) and seven GO terms (fat cell differentiation, calcium ion binding, cytoplasm, nucleus, phospholipid transport, central nervous system development, and cell surface) that were found to be shared among all the traits. Our data provide insights into the genes and pathways associated with obesity and obesity-related traits.

## 1. Introduction

Canine obesity is a global epidemic rising all over the world. Among dogs visiting veterinary practices, roughly 34–59% of dogs are reported to be overweight, with 5–20% obese [1,2,3,4]. The contemporary rise in obesity is triggered by lifestyle; however, the extensive inter-individual deviation in body mass index (BMI) witnessed even under shared environmental conditions can only be attributed to a genetic predisposition to the condition [5]. Genetic mutations within a single gene have been reported to have a large effect on obesity [6]. Inbreeding leading to reduced genetic diversity is also considered as a crucial factor associated with obesity. Inbreeding increases the prevalence of genetic defects because of the homozygosity of a large number of deleterious recessive alleles [7].

In addition, as in human beings, obesity causes several adverse consequences in dogs, such as cardiopulmonary disease, insulin resistance, diabetes, osteoarthritis, cancer, and other endocrine disorders [4,8,9]. Canines are considered to be obese when their body weight exceeds 30% of their optimal body weight [4]. Gaining extra body weight always precedes the onset of obesity [4]. In obese individual, adipocytes release elevated amounts of free fatty acids, cytokines (TNF-α), leptin, glycerol, adiponectin, and several other factors that are involved in regulating insulin secretion, blood sugar level and body weight, which ultimately contributes to the development of insulin resistance [10,11,12]. Previously, many studies have reported that body weight, obesity, and blood sugar have a clear positive association [13,14,15]. The dog is also considered an important model for obesity research because of its clinical and molecular similarity with humans, but the knowledge about the genetic contribution to dog obesity remains insufficient.

Post-genome-wide association study (GWAS) analyses have been performed in several studies to discover obesity-related markers [16,17,18]. Previous association studies have revealed that polymorphism of *POMC* and *TNF* gene can affect the body weight and appetite in obesity [16,19]. A few other genes such as *FTO*, *PPARG*, *BDNF*, *MC4R,* and *MC3R* have been reported to be associated with obesity in canines [16,17,18]. Till now, only a few studies on the effect of polymorphism of canine genes on obesity have been published. On the other hand, polymorphism of more than 50 genes was reported to be associated with human obesity [4,20]. Therefore, the continuous search for important candidate canine genes associated with obesity is required. It will help to understand the etiology of obesity in canine. GWAS is a widely used method for studying the genetics of complex diseases [21]. GWAS have so far yielded a huge number of associations between single-nucleotide polymorphisms (SNPs) and complex diseases [21,22,23]. However, performing GWAS powerful enough is relatively difficult because of a few factors like small effect sizes of variants in complex disease, and required stringent statistics because of multiple testing problems. As a result, GWAS identifies only large effect sizes of variants [24]. In addition, GWAS does not capture multi-allelic QTL because of the bi-allelic nature of single nucleotide variants (SNVs) and does not account the fact that disease-associated genes or their products work together in a network [22,23,24]. Therefore, a robust GWAS analysis with the aim of detailed biological understanding is challenging. The solutions proposed to overcome these limitations are to focus on post-GWAS analyses such as functional gene and pathway enrichment analysis, protein–protein interaction network analysis, and translational medicine [22,24,25,26].

In the present study, we performed a case-control-based GWAS analysis using SNP genotyping data from Illumina CanineHD BeadChip array and supplemented it with gene-set and pathway-based functional analysis to detect significantly associated candidate genes and pathways with obesity, and related traits (blood sugar and body weight) in dogs.

## 2. Materials and Methods

### 2.1. Animals and Phenotype Assignment

A total of 153 dogs from across South Korea were used in the present study and blood samples were collected from them by following relevant guidelines formulated by the Institutional Animal Care and Use Committee (IACUC) of the National Institute of Animal Science (NIAS, RDA, Wanju-gun, South Korea), and protocol consent was obtained for ‘Development of early diagnosis technology for degenerative muscular skeleton system in special-purpose dog’ project. All dogs were diagnosed individually by a veterinarian. Obesity traits were classified into a 5-point scoring system (1–5) using the body condition score (BCS) [27] as given in Table 1. BCS scores of 1 to 4 were used as controls and a score of 5 as a case for the case-control analysis of obesity. For case-control analysis of body weight trait, the dog of a particular breed having higher body weight, higher than the threshold suggested in America Kennel club standards, was selected as case and dogs falling within the suggested standards [28,29] were considered as control. The case–control analysis for blood sugar was distinguished based on the blood glucose concentration. Individuals with fasting blood glucose level over 120 mg/dL were considered as case. Dogs whose fasting blood glucose level is in the normal range (80−120 mg/dL) were considered as control. All dogs were kept under fasting for 12 h from their last meal before the blood sampling in the morning.

### 2.2. Genomic DNA Extraction, SNP Genotyping and Quality Control

Genomic DNA was extracted from blood samples of 153 dogs of 18 different breeds, using the DNeasy Blood and Tissue Kit (Qiagen, Valencia, CA, USA). The samples were genotyped on an Illumina CanineHD BeadChip (Illumina, San Diego, CA, USA) array, which contains 173,662 SNPs. Quality control (QC) was carried out with PLINK v.1.9 software [24] under the following criteria: minor allele frequency <5%, low genotyping call rate <90%, missing genotype calls >10%, Hardy–Weinberg equilibrium at *p* < 0.000001. The final genotyping call rate was 98.5%. Following QC filtering, 135,553 SNPs and 152 animals remained for further association analysis.

### 2.3. Genome-Wide Association Analysis

A genome-wide association study (GWAS) was performed using a logistic regression model implemented in PLINK v.1.9, to test the association between disease trait and allele in each SNP. Significant factors such as age (1–17), sex (85 females 68 males), and breed (18) were fitted in the GWAS statistical model for all the traits. We generated a total of 20 principal components (PCs); the eigenvalues of all the PCs were fit as co-variance to account for population stratification. The GWAS statistical model used was as follows:

*y* = *xβ* + *zu* + *e* (1)

where *y* is a phenotype, *x* and z are design matrices, *β* is a vector of fixed effects, u is an additive genetic effect for each marker, and e is a vector of residual [30]. A Bonferroni-corrected threshold was applied to correct for multiple testing, and the genome-wide significance threshold was *p* < 3.74 × 10^−7^ (~0.05/135,553). Manhattan and quantile-quantile (Q-Q) plots were generated using CMplot package in R [24].

### 2.4. Gene-Set Enrichment and Pathway Analysis

We conducted gene-set enrichment and pathway analysis for each trait following the methods described by Dadousis et al. [24,25]. The markers were assigned to harboring genes or to genes within a flanking region of 5 kb up- and downstream of the SNP using SnpEff version 4.3 software [24]. A nominal *p* < 0.01 was used to filter for SNPs from the GWAS analysis for gene-set enrichment and pathway analysis. The reason behind adopting a less stringent threshold for GWAS was to identify SNPs which do not reach the stringent significant thresholds set in GWAS analysis but still contributes to phenotypic variability. Furthermore, gene-set and pathway analyses obtained from GWAS provided additional knowledge about the complex relationships among genes and interrelated pathways which are likely to play role in obesity [31]. Merging less significant but connected SNPs we can understand well how these variants might be collectively related to our phenotypes of interest [31]. The SNP IDs, genes name assigned to SNPs was filtered from a variant call format (VCF) file that was previously mapped using SnpEff version 4.3 software. The Kyoto Encyclopedia of Genes and Genomes (KEGG) pathway database and Gene Ontology (GO) database under biological process and molecular function categories were used for functional annotation and enrichment analyses. Over-representation of genes in every GO terms and pathways were tested using a Fisher’s exact test, which was conducted using the web-based gene-enrichment analysis tool, the Database for Annotation, Visualization, and integrated discovery (DAVID, http://david.abcc.ncifcrf.gov/) [32]. False discovery rate (FDR) correction (<0.05) was applied to account for multiple testing [32]. The GO terms/KEGG category pathways with more than 10 and less than 1000 genes were analyzed for narrowing down the functional categories.

## 3. Result and Discussion

A polygenic complex disease such as obesity is generally controlled by additive genetic effects of a large number of genes which harbors several SNPs with small-effect size [24]. Some of these SNPs do not reach the stringent statistic thresholds, which are adapted to control for multiple testing problems in GWAS. Therefore, in this study, we included GO and pathways enrichment analyses, which were performed using the post-GWAS assigned genes (harboring essential SNPs) to understand the genetic contribution and biological significance of the functional SNPs on three traits (obesity, blood sugar, and body weight).

### 3.1. Phenotypes

In the current study, 85 females and 68 males between 1 and 17 years of age were used for analysis. As shown in Table 1, a total of 153 dogs are classified into 5 different BCS categories for case/control analysis of obesity trait. The dogs with a BCS score of 5 were designated as obese [27]. Therefore, we assigned this dog group as a case. In total, there were 29 cases and 124 controls for obesity trait. The total number of cases and controls for body weight was 28 and 125. The overlap between the two categories was 28 individuals, which were both obese and cases for body weight. For blood sugar, it was 34 cases and 119 controls (Table 2). Earlier studies have reported that the medium-sized dogs are more likely to be obese than small-sized dogs or toy breed dogs [27,33]. In this study, most of the obese animals were also from the small category dogs (Pomeranian, Yorkshire Terrier, and Miniature Pinscher) or toy-sized (Maltese and Shihtzu) (Table 3). On the other hand, only a few cases were medium-sized dogs such as Beagles and Cocker Spaniel. Possibly, the small number of medium and large-sized dogs in our study is the reason for inconsistent result with the previously reported study [27,33].

### 3.2. Genome-Wide Association Study

**Figure 1:**
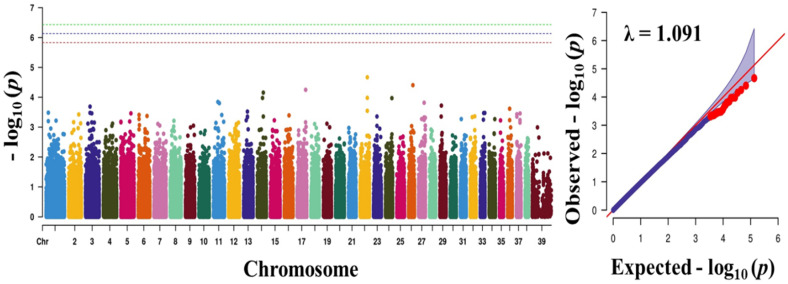
Manhattan plots showing the distribution of *p*-values of single nucleotide polymorphism (SNP) markers associating with obesity. Greenline designates the genome-wide significant threshold level of *p* < 3.74 × 10^−7^, blue line designates suggestive threshold level of *p* < 7.48 × 10^−7^, and red line designates suggestive threshold level of *p* < 5.82 × 10^−6^. The quantile-quantile (Q-Q) plot of the GWAS is shown on the right side. GWAS—genome-wide association study.

**Figure 2:**
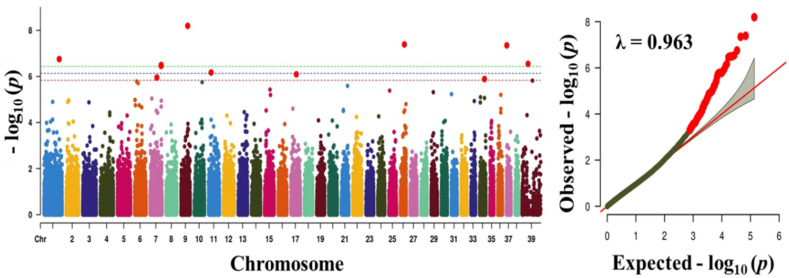
Manhattan plot showing the distribution of *p*-values of single nucleotide polymorphism (SNP) markers associated with body weight. Greenline designates the genome-wide significant threshold level of *p* < 3.74 × 10^−7^, blue line designates suggestive threshold level of *p* < 7.48 × 10^−7^, and red line designates suggestive threshold level of *p* < 5.82 × 10^−6^. The quantile-quantile (Q-Q) plot of the GWAS is shown on the right side. GWAS - genome-wide association study.

**Figure 3:**
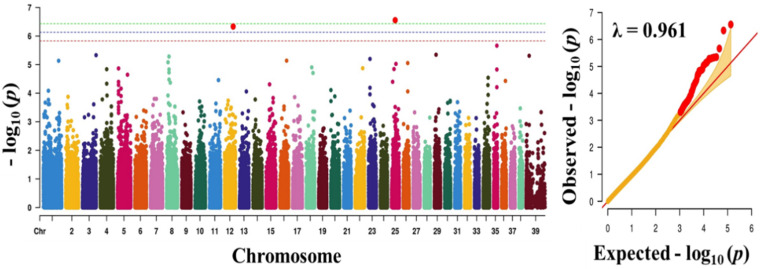
Manhattan plot showing the distribution of *p*-values of single nucleotide polymorphism (SNP) markers associating with blood sugar. Greenline designates the genome-wide significant threshold level of *p* < 3.74 × 10^−7^, blue line designates suggestive threshold level of *p* < 7.48 × 10^−7^, and red line designates suggestive threshold level of *p* < 5.82 × 10^−6^. The quantile-quantile (Q-Q) plot of the GWAS is shown on the right side. GWAS - genome-wide association study.

To find significant loci associated with the three traits (obesity, body weight, and blood sugar), we performed GWAS with 133,553 SNPs which passed our filtering criteria. The Q-Q plot of the association study indicated a significant clear deviation for blood sugar (λ value = 0.961) and body weight (λ value = 0.963), but, for obesity, there was no obvious deviation (λ value = 1.091) (Figure 1, Figure 2 and Figure 3). The *p*-value obtained from the GWAS was plotted as Manhattan plots (Figure 1, Figure 2 and Figure 3). Our result showed that 2 and 7 SNPs were significantly associated to blood sugar and body weight, respectively, at the genome-wide significance level of *p* < 3.74 × 10^−7^ (i.e., Bonferroni corrected threshold (0.05/133,553). The candidate SNPs associated with blood sugar were on chromosomes 25 and 12; whereas for body weight they were on chromosome 9, 26, 37, 1, 39, and 7 (Table 4). Moreover, none of the SNPs were found to be significant (*p* < 3.74 × 10^−7^) for obesity.

The two SNPs significantly associated with blood sugar, BICF2P1418953 (downstream gene variant) and BICF2G630121162 (intragenic region) were annotated to *PRSS55* and *GRIK2* genes, respectively (Table 4). The serine protease 55 (*PRSS55*) belongs to the member of a group of membrane-anchored chymotrypsin (S1)-like serine protease that has been proven to play a key role in maintaining homeostasis [34,35,36]. Obesity is directly attributable to homeostasis imbalance [37]. Furthermore, glutamate receptor ionotropic receptor kainite type subunit 2 (*GRIK2*) is involved in the majority of glutametergic neurotransmission, which has been suggested to take part in the onset of obesity by regulating appetite [38,39].

In addition, BICF2P1168261 (intron variant), G1314f25S201 (synonymous variant), BICF2P940718 (intergenic region), BICF2P407675 (intron variant), BICF2P1124008 (intergenic region), and BICF2S23242598 (intron variant), which were significantly associated with body weight, were located in protein-coding genes Calcium Voltage-Gated Channel Subunit Alpha1 B (*CACNA1B*), *U6* (U6 non-coding small nuclear RNA), Myosin Heavy Chain 14 (*MYH14*), Protein Tyrosine Phosphatase Non-receptor Type 2 (*PTPN2*), and SEH1 like Nucleoporin (*SEH1L*), respectively. The most significantly associated amongst these was BICF2P116826, harbored by calcium voltage-gated channel subunit alpha1 B (*CACNA1B*), a member of the voltage-gated calcium channels [40]. The voltage-gated calcium channels are the main regulators of calcium signaling in adipogenesis, which eventually contribute to obesity [40,41,42]. In addition, *CACNA1B* gene has been previously reported to be a therapeutic target for diseases such as lung cancer, prostate cancer and breast cancer [40]. Moreover, the gene, *CACNA1B* was noticed to be significantly enriched in several other pathways associated with the traits evaluated in this study, suggesting a significant connection with obesity disease. Therefore, we suggest that the *CACNA1B* gene can be a good candidate marker. The body weight trait in dogs has been examined in several studies based on the GWAS approach [29]. Though no previously reported genes/markers were found to be associated in our study, some of the candidate markers/genes identified in this study were on chromosome X (indicated as chr 39 in this study), which was previously reported to harbor genes/markers found to have a significant effect on body weight in dogs [29]. The lack of overlapping genes/marker with previous reports may be due to use of different breeds in this study, and the inclusion of higher number of large breed dogs, and differences in phenotype assignment.

In addition, compared to obesity in humans, there are only limited reports on genetic markers associated with canine obesity. In humans, more than 220 obesity-related genetic markers have been documented throughout the last decade [16]. Among these, *FTO*, *BDNF*, *MC4R*, *PCSK1*, *MC3R*, and *PPARG* genes were considered as promising candidate genes for canine obesity [16,17,18,43]. Recently, Mankowska et al. reported another candidate gene, *POMC,* which showed a strong association with weight and appetite in obesity-prone Labrador retrievers [19,20]. In the current study, several different candidate genes were found; however, none were common to those previously reported. However, the identified markers could serve as a good candidate for further studies.

### 3.3. Gene-Set Enrichment and Pathway Analysis

GWAS was complemented with a functional enrichment analysis to explore the significant pathways/GO terms associated with obesity and other traits. It showed the enrichment of annotated gene-sets which worked together in a network to carry out specific molecular processes. Out of 135,554 SNPs used in GWAS, 91,779 were located within mapped genes or the 5 Kb flanking regions of the mapped genes. A total of 1057, 1409, and 1243 SNPs showed an association for obesity, body weight, and blood sugar at a nominal threshold of *p* < 0.01, respectively (Supplementary Table S1). These SNPs were mapped to 449, 933, and 820 unique genes for obesity, body weight, and blood sugar trait, respectively (Supplementary Table S1). Subsequently, gene-set and pathway analysis were performed using DAVID. A total of 84, 114, and 120 GO terms and 21, 44, and 24 KEGG pathways were identified to be enriched for obesity, blood sugar, and body weight, respectively (Supplementary Table S2). Out of the total enriched GO terms and KEGG pathways, the top 5 significantly enriched GO terms and pathways are presented in Table 5. The pathways commonly enriched in all the three traits (obesity, blood sugar, and body weight) (Table 6) were: cfa04310—Wnt signaling pathway, cfa04520—adherens junction, cfa05200—pathways in cancer, cfa04360—axon guidance, and cfa04911—insulin secretion. Likewise, a total of 7 GO terms (GO:0045444—fat cell differentiation, GO:0005509—calcium ion binding, GO:0005737—cytoplasm, GO:0005634—nucleus, GO:0015914—phospholipid transport, GO:0007417—central nervous system development, GO:0009986—cell surface) were found to be commonly enriched in all the three evaluated traits (Table 6). 

#### 3.3.1. Wnt Signaling, Adherens Junction, and Axon Guidance Pathways

Wnt signaling is a crucial pathway for adipogenesis. This pathway is involved in the development of obesity-induced insulin resistance [44,45]. Wnt signaling has been proposed as a targeting pathway to fight obesity due to its critical role in the development of white adipose tissue and brown adipose tissue [46]. Moreover, an important transcription factor of Wnt signaling was previously identified as a candidate gene for type 2 diabetes [47]. In addition, adherens junction and axon guidance were significantly enriched in obesity, blood sugar, and body weight. An adherens junction is an intercellular junction and is important for epithelial adhesion. Adherens junction plays a key role in the regulation of insulin secretion using cadherin protein which ultimately reveals strong association with blood glucose level, obesity, and diabetes [48]. Amongst the enriched genes in these pathways, Wnt family member 1 (*WNT1*) was previously reported to be associated with obesity and the importance of this gene in obesity was also highlighted [46,49].

#### 3.3.2. Cancer-Related Pathways

The pathways in cancer (cfa05200) were significantly enriched in all the three traits including obesity, blood sugar, and body weight. Apart from that other significantly enriched cancer-related pathways, including, endometrial cancer (cfa05213), melanoma (cfa05218), prostate cancer (cfa05215), proteoglycans in cancer (cfa05205) were detected with some pathways being in common among all traits. The association between cancer and obesity is well documented, obesity increases the risk of various cancers in the human population [50,51]. Further, Lim et al. reported that the increased expression of obesity-associated molecules increases the risk of development of mammary gland tumors in obese female canines [51]. These pathways are enriched with numerous genes and in fact, the mutations in a few key genes have been described to affect obesity or blood sugar trait [12,49,52,53,54]. In total, 64 genes were significantly enriched in cancer-related pathways and several significant genes such as cyclic AMP-responsive element-binding protein 5 (*CREB5*) [55], AKT serine/threonine kinase 3 (*AKT3*) [53], cyclin D1 (*CCND1*) [12,52], Wnt family member 1 (*WNT1*) [49,54] were identified to be associated with blood sugar and obesity traits.

#### 3.3.3. Insulin Secretion Pathway

Another important pathway enriched with genes harboring SNPs associated with obesity was insulin secretion (cfa04911). This pathway was enriched with 7 genes in total. Insulin secretion occurs in pancreatic *β* cells and it triggers the fusion of insulin-containing granules with the cell membrane [10]. Insulin secretion is an integral part of blood glucose levels control system. [10]. The positive association between obesity and insulin has been investigated for decades [10,11]. Obesity-associated with insulin resistance is a major risk factor of type 2 diabetes. However, insulin also plays a vital role in obesity by inducing ATP production in mitochondria to prevent 5՛ AMP-activated protein kinase (AMPK) activity during hyperinsulinemia condition [56]. Recently, an important link between the insulin level and obesity was reported: diet-induced hyperinsulinemia assisted by glucagon hormone is an obligatory factor for obesity [11].

#### 3.3.4. Other Enriched Gene Ontology Terms

We have highlighted seven important GO terms, which were significantly enriched in all three traits. These included fat cell differentiation, calcium ion binding, cytoplasm, nucleus, phospholipid transport, central nervous system development, and cell surface. The fat cell differentiation or adipocyte formation plays a crucial role in adipogenesis during obesity [46]. Calcium is an important ion for normal physiological functioning and calcium ion binding is involved in calcium signaling pathways which have been shown as key pathways in the regulation of obesity in many studies [40,41,42,57]. The multiple calcium signaling pathways play a most important role in the biological clock, neuronal excitability, and intestinal microbial activity for regulating the food intake and adipocyte metabolism, which subsequently reduces the occurrence of obesity [57]. The genes enriched included CACNA1B, which also harbored the significantly associated SNP BICF2P116826, encoding calcium voltage-gated channel subunit alpha1 B, which is associated with adipogenesis [40,41,42] and most importantly, it was identified as a candidate gene for body weight trait in our association study. Furthermore, the relation of phospholipid transport and obesity has been suggested to play a vital role in lipoprotein metabolism by transferring the phospholipids from triglyceride-rich lipoproteins to high-density lipoproteins [58].

## 4. Conclusions

The present study is the first to report about a post-GWAS analyses approach to prioritize the identification of genetic loci, pathways, and genes underlying molecular mechanisms of canine obesity and related traits such as body weight and blood sugar in multi-breed dogs. We have identified several significant candidate genes associated with obesity-related traits; in particular, CACNA1B gene harboring SNP BICF2P116826 could be a possible candidate gene for canine obesity. The gene-set and pathway analyses revealed five shared pathways, (Wnt signaling pathway, adherens junction, pathways in cancer, axon guidance, and insulin secretion) and seven GO terms to be associated with all evaluated traits, which probably explain that obesity is a polygenic trait. Overall, our results provide clues for identification of candidate SNPs and genes which have a significant impact on the etiology of canine obesity.

## Figures and Tables

**Table 1:** Total dogs categorized based on body condition score for obesity case–control analysis.

Body Condition Score	1	2	3	4	5 (Case)
	Very thin	Underweight	Ideal body weight	Overweight	Obese
Number of animal	7	11	98	11	29

**Table 2:** Number of cases and controls used in this study for each trait.

Group	Number of Animals	Number of Control	Number of Case
Obesity	153	124 (BCS 1–4)	29 (BCS 5)
Body weight	153	125	28 *
Blood sugar	153	119 (≤120 mg/dL)	34 (≥120 mg/dL)

* Dogs weighing over the America Kennel club standards were selected as cases; BCS—body condition score.

**Table 3:** Summary of phenotypes (including breed, number of male and female dogs investigated, and number of cases) used to perform GWAS in our analysis.

No.	Breed Name	Number of Dogs Investigated	Obesity	Body Weight	Blood Sugar
		85 Females 68 Males	Case	Case	Case
1	Beagle	3	1	1	1
2	Bichon fris	1	0	0	0
3	Chihuahua	6	0	0	2
4	Cocker Spaniel	8	1	1	1
5	Dachshund	3	0	0	0
6	Doberman	3	0	0	1
7	German Shepherd	1	0	0	1
8	Golden Retriever	1	0	0	0
9	Maltese	40	8	7	2
10	Miniature Pinscher	3	5	5	7
11	Mixed	19	1	1	1
12	Parson Russell Terrier	5	5	5	3
13	Pomeranian	7	2	2	6
14	Poodle	20	1	1	2
15	Schnauzer	6	1	1	1
16	Shih tzu	10	2	2	2
17	Spitz	5	0	0	0
18	Yorkshire Terrier	12	2	2	4

**Table 4:** Top SNPs associated with obesity, body weight, and blood sugar in dogs.

Trait	SNP ID	Chr	Position	Freq	Gene	Type
Body weight	BICF2P1168261	9	47831552	6.38 × 10^−9^	*CACNA1B*	Intron variant
	G1314f25S201	26	29766195	4.09 × 10^−8^	*C22orf39*	Synonymous variant
	BICF2P940718	37	5443556	4.50 × 10^−8^	*U6*	Intergenic region
	BICF2P407675	1	1.06 × 10^8^	1.77 × 10^−7^	*MYH14*	Intron variant
	BICF2P247463	39	39876923	2.86 × 10^−7^	-	-
	BICF2P1124008	7	78278707	3.20 × 10^−7^	*PTPN2*	Intergenic region
	BICF2S23242598	7	78365053	3.20 × 10^−7^	*SEH1L*	Intron variant
Blood sugar	BICF2P1418953	25	27416887	2.78 × 10^−7^	*PRSS55*	Downstream_gene_variant
	BICF2G630121162	12	60529545	4.64 × 10^−7^	*GRIK2*	Intergenic_region

**Table 5:** Top 5 Gene Ontology and KEGG pathways significantly enriched using genes associated with obesity, body weight, and blood sugar.

Trait	Category	Term_ID	Term	Count	%	*p*-Value	Genes
Obesity	KEGG_PATHWAY	cfa04360	Axon guidance	10	0.015278	7.15 × 10^−4^	*DCC, MAPK1, NGEF, EPHA7, CXCR4, GNAI1, ROBO1, UNC5D, LRRC4C, EPHB1*
	KEGG_PATHWAY	cfa04550	Signaling pathways regulating pluripotency of stem cells	10	0.015278	0.0015	*MAPK1, FGFR1, BMP2, ONECUT1, FZD1, FZD3, WNT11, FZD2, ZFHX3, KLF4*
	KEGG_PATHWAY	cfa05200	Pathways in cancer	18	0.0275	0.0016	*DCC, FGFR1, BMP2, COL4A1, BRAF, GNAI1, FGF9, RUNX1T1, FZD1, FZD3, FZD2, GLI3, CTNNA3, CTNNA2, LAMA2, MAPK1, CXCR4, WNT11*
	KEGG_PATHWAY	cfa04520	Adherens junction	7	0.010695	0.0026	*MAPK1, FGFR1, TJP1, PTPRM, SSX2IP, CTNNA3, CTNNA2*
	KEGG_PATHWAY	cfa05217	Basal cell carcinoma	6	0.009167	0.0040	*BMP2, FZD1, FZD3, WNT11, FZD2, GLI3*
	GOTERM_MF_DIRECT	GO:0016874	Ligase activity	7	1.682692	3.74 × 10^−4^	*HECW2, UBE3A, SUCLG2, HECTD3, SIAH1, SMURF1, NEDD4L*
	GOTERM_BP_DIRECT	GO:0045892	Negative regulation of transcription, DNA-templated	13	3.125	0.0031	*RBFOX2, BCLAF1, RUNX1T1, FZD1, PAX2, CBFA2T3, ADIPOQ, GAS6, ZSCAN10, LHX1, ATP8B1, POU3F3, WNT11*
	GOTERM_BP_DIRECT	GO:0060022	Hard palate development	3	0.721154	0.0034	*FZD1, FZD2, MMP25*
	GOTERM_BP_DIRECT	GO:0034115	Negative regulation of heterotypic cell-cell adhesion	3	0.721154	0.0056	*APOA1, ADIPOQ, KLF4*
	GOTERM_BP_DIRECT	GO:0002062	Chondrocyte differentiation	5	1.201923	0.0057	*SNX19, FGFR1, BMP2, FGF9, NFIB*
Body weight	KEGG_PATHWAY	cfa05033	Nicotine addiction	8	0.900900	0.0021	*GABRG3, GRIA2, GRIA1, GABRB1, GABRA5, GRIN2A, GRIN3A, CACNA1B*
	KEGG_PATHWAY	cfa04080	Neuroactive ligand-receptor interaction	23	2.590090	0.0049	*GABRG3, GLRA1, GRIK2, GABRB1, OPRK1, GRIN3A, P2RY6, GRIA2, GRIA1, NMUR2, HRH4, ADRA1A, NMBR, PRL, CHRNE, PTAFR, GRID1, GHR*
	KEGG_PATHWAY	cfa05206	MicroRNAs in cancer	13	1.463963	0.0268	*KIF23, PDGFA, SOCS1, MET, BMPR2, PIM1, TP63, ZEB1, IRS1, PDCD4, CCND1, CDKN2A, DNMT1*
	KEGG_PATHWAY	cfa04022	cGMP-PKG signaling pathway	14	1.576576	0.0293	*EDNRA, KCNU1, KCNMB4, PLCB4, TRPC6, ATP2A3, GTF2IRD1, ADRA1A, CREB5, NOS3, PLCB1, CACNA1D, IRS1, KCNMB2*
	KEGG_PATHWAY	cfa04310	Wnt signaling pathway	12	1.351351	0.0394	*DKK2, MAP3K7, CCND1, PLCB4, DKK1, VANGL1, PRICKLE1, MMP7, SIAH1*
	GOTERM_MF_DIRECT	GO:0004725	Protein tyrosine phosphatase activity	15	1.689189	5.27 × 10^−5^	*PTPRB, CDC14A, PTPN2, CDC14B, EPM2A, DUSP10, PTPN13, PTPRT, PTPRU, EYA3, EYA4, EYA1, DUSP26, UBASH3B, PTPN1*
	GOTERM_BP_DIRECT	GO:0045444	Fat cell differentiation	12	1.3513513	1.90 × 10^−4^	*BBS2, METTL8, CCND1, FAM120B, SMAD6, BBS9, FFAR2, SOCS1, OSBPL11, TTC8, PIAS1, PLCB1*
	GOTERM_CC_DIRECT	GO:0005794	Golgi apparatus	43	4.8423423	3.54 × 10^−4^	*GLIS3, ACHE, SYT4, RAB3GAP1, NOS3, CDK5RAP2, JAKMIP2, OLFM3, GOLM1, TERF2, CDK13, KLF5, NMNAT2, MSH6, CLN3, PLD1, MYO6, DNM1L, CCDC88A, LYN, ACO1, BEND5, GOLIM4, PKDCC, NMT2, ATF6, DUSP26, CPE, BACE2, SULF1, DYM, RAB14, SGCE, CWC22, EXT1*
	GOTERM_BP_DIRECT	GO:0007156	Homophilic cell adhesion via plasma membrane adhesion molecules	14	1.5765765	4.51 × 10^−4^	*CADM1, CLSTN2, SDK2, PCDH15, PCDH17, CDH8, CDH13, DSG2, CDH18, FAT1, CDH19, FAT2, CDH26, KIRREL3*
	GOTERM_CC_DIRECT	GO:0043025	Neuronal cell body	16	1.8018018	7.20 × 10^−4^	*GLRA1, GRIK2, DENND1A, GDPD5, GRIN3A, KLHL1, ALCAM, SEZ6L2, APOB, BRINP1, CPE, GRIA1, PSEN2, RAPGEF2, BRINP3, CACNA1B*
Blood sugar	KEGG_PATHWAY	cfa04919	Thyroid hormone signaling pathway	14	1.837270341	8.07 × 10^−4^	*ACTB, THRB, ATP1A1, RCAN2, PLCB3, CCND1, PLCB4, DIO2, GSK3B, PLCG2, PRKACB, PLCB1, AKT3, PIK3R1*
	KEGG_PATHWAY	cfa05206	MicroRNAs in cancer	15	1.968503937	0.0019	*IRS2, E2F3, MCL1, MMP16, CDK6, ZEB2, ZEB1, PRKCE, TIMP3, RPS6KA5, CCNE1, CCND1, PLCG2, DNMT1, ZFPM2*
	KEGG_PATHWAY	cfa05223	Non-small cell lung cancer	9	1.181102362	0.0023	*FHIT, CCND1, E2F3, PLCG2, CDK6, EGF, AKT3, PIK3R1, EML4*
	KEGG_PATHWAY	cfa04916	Melanogenesis	12	1.57480315	0.0026	*WNT1, PLCB3, PLCB4, GSK3B, MITF, EDN1, FZD1, KITLG, PRKACB, PLCB1, WNT7A, CALM1*
	KEGG_PATHWAY	cfa04750	Inflammatory mediator regulation of TRP channels	12	1.57480315	0.0031	*PLCB3, IL1R1, PLCB4, PLCG2, IL1RAP, PLA2G6, PRKCH, PRKACB, PRKCE, PLCB1, PIK3R1, CALM1*
	GOTERM_MF_DIRECT	GO:0004222	Metalloendopeptidase activity	15	1.968504	0.0011	*ELP3, IRS2, BMP2, LYN, EDN1, HGF, DAB2, SEMA6D, FOXF1, SEMA3C, COL1A1, PDGFD, PAK1, PIK3R1, CSF1R*
	GOTERM_BP_DIRECT	GO:0030335	positive regulation of cell migration	31	4.068241	0.0017	*FRK, THRB, MITF, EDN1, ZEB2, PRDM16, EPC1, REL, FOXF1, PRMT6, ETV6, DLG1, SIM2, ALX1, TBL1XR1, BMP2, ASXL1, ZHX2, LMCD1, SMYD2, SHOX2, HDAC4, CCND1, DKK1, DUSP26, PDE2A, HOPX, TFAP2B, DNMT1, RIPPLY2, BMP6*
	GOTERM_BP_DIRECT	GO:0000122	negative regulation of transcription from RNA polymerase II promoter	27	3.543307	0.0017	*CYB5R4, CAV2, GALNT1, RAB3C, PKHD1, SLC39A12, PINK1, ARFGEF1, SLC11A2, APP, BDNF, ECE1, PTK2B, FAT1, TMEM192, CDK5RAP2, DLG1, PTPRM, LYN, STC2, PRKCE, PDE2A, VAMP8, GSK3B, CYFIP2, AKAP6, SPAST*
	GOTERM_CC_DIRECT	GO:0048471	perinuclear region of cytoplasm	11	1.44357	0.0039	*EPHA5, SEMA6A, ZNF280D, KIF5B, ANK3, ROBO1, SEMA3C, ETV1, RELN, UNC5D, CSF1R*
	GOTERM_BP_DIRECT	GO:0007411	axon guidance	8	1.049869	0.0041	SOX10, EDN3, SEMA6A, SEMA6D, SEMA3C, KITLG, ZEB2, ALX1

**Table 6:** The significantly enriched GO terms and KEGG pathways shared amongst obesity, body weight, and blood sugar.

Category	Term_ID	Term	Genes ^a^
GOTERM_BP_DIRECT	GO:0045444	Fat cell differentiation	*AQP1*
GOTERM_MF_DIRECT	GO:0005509	Calcium ion binding	*CACNA1B, GRIK2*
GOTERM_CC_DIRECT	GO:0005737	Cytoplasm	*ROBO1, LDHB*
GOTERM_CC_DIRECT	GO:0005634	Nucleus	*ATP2B1, CSRNP3, CSRNP2*
GOTERM_BP_DIRECT	GO:0015914	Phospholipid transport	*PCTP*
GOTERM_BP_DIRECT	GO:0007417	Central nervous system development	*CHD7*
GOTERM_CC_DIRECT	GO:0009986	Cell surface	*IL1R1, ROBO1, WNT1*
KEGG_PATHWAY	cfa04310	Wnt signaling pathway	*WNT11, MAP3K7, WNT7A*
KEGG_PATHWAY	cfa04520	Adherens junction	*MAPK1, CTNNA2, MAP3K7*
KEGG_PATHWAY	cfa05200	Pathways in cancer	*CCND1, FZD1, FZD3, FZD2, CTNNA1*
KEGG_PATHWAY	cfa04360	Axon guidance	*MAPK1, ROBO1*
KEGG_PATHWAY	cfa04911	Insulin secretion	*CACNA1B, CACNA1D, PLCB4*

^a^ Enriched genes in KEGG pathways and GO shared among three traits, GO—Gene Ontology, KEGG—Kyoto Encyclopedia of Genes and Genomes.

## Supplementary Materials

The following are available online at https://www.mdpi.com/2076-2615/10/11/2071/s1, Table S1: List of all SNPs (*p* < 0.01) associated with obesity, body weight, and blood sugar that were used for gene-set and pathway analysis, Table S2: List of all Gene Ontology and KEGG pathways significantly enriched using genes associated with obesity, body weight, and blood sugar.
